# Raltitrexed-Modified Gold and Silver Nanoparticles for Targeted Cancer Therapy: Cytotoxicity Behavior In Vitro on A549 and HCT-116 Human Cancer Cells

**DOI:** 10.3390/ma14030534

**Published:** 2021-01-22

**Authors:** Jeroni Morey, Pere Llinás, Alberto Bueno-Costa, Alberto J. León, M. Nieves Piña

**Affiliations:** 1 Department of Chemistry, University of the Balearic Islands, Crta. de Valldemossa, Km. 7.5, 07122 Palma de Mallorca, Balearic Islands, Spain; jeroni.morey@uib.es (J.M.); albertoperezleon@hotmail.com (A.J.L.); 2 Department of Biochemistry, University of the Balearic Islands, Crta. de Valldemossa, Km. 7.5, 07122 Palma de Mallorca, Balearic Islands, Spain; pllinasarias@gmail.com (P.L.); abueno@carrerasresearch.org (A.B.-C.)

**Keywords:** gold nanoparticles, silver nanoparticles, raltitrexed, cancer therapy

## Abstract

Two different raltitrexed gold and silver nanoparticles for the delivery of an antitumoral drug into cancer cells were synthesized and characterized. A cysteine linker was used for the covalent bonding of raltitrexed to the surface of nanoparticles. To evaluate the efficacy of the antifolate-derivative nanoparticles, their cytotoxicity was assayed in vitro with A549 human lung adenocarcinoma and HCT-116 colorectal carcinoma human cells. Modified nanoparticles are a biocompatible material, and administration of silver raltitrexed nanoparticles strongly inhibited the viability of the cancer cells; gold raltitrexed nanoparticles do not show any type of cytotoxic effect. The results suggest that silver raltitrexed nanoparticles could be a potential delivery system for certain cancer cells.

## 1. Introduction

Covalently functionalized inorganic nanoparticles (NPCs) with antitumor drugs are potentially a useful tool in the treatment of some types of cancers [1,2]. NPCs have been used in medicine and biology for the transport and delivery of drugs as well as in gene therapy, immunotherapy [3], magnetic resonance imaging (MRI) [4,5], metabolic pathway signaling, photodynamic therapy (PDT), and studies of drug accumulation [6,7]. Although the cytotoxicity of inorganic nanoparticles (NPs) is still being reviewed, factors such as the chemical nature of the nanoparticle (NP), size, shape, and surface charge can affect toxicity. The toxicity of NPs in general terms is often mediated by the formation of reactive oxygen species or by the denaturation of DNA [8] or proteins. Although nanoparticles of gold [9,10,11], (AuNPs), silver (AgNPs) [8,9,12], magnetite Fe_3_O_4_NPs [13], and silica SiO_2_NPs [12,14] are generally considered to be good transport vectors and biocompatible, they continue to be investigated for their use in anticancer therapy [15].

Raltitrexed is known commercially as Tomudex or ZD1694. It is an anticancer drug from the quinazoline antifolate family and has been used as a chemotherapeutic agent against colorectal cancer since 1996 [16]. It has a similar structure to *N*5, *N*10-methylenetetrahydrofolate (5,10-CH2-THF) a derivative of folic acid (Vitamin B9). This antifolate is a potent mixed non-competitive inhibitor of the enzyme thymidylate synthase (TS) presenting a Ki of 62 nM [17]. This enzyme is responsible for synthesizing thymidylate (dTMP) from deoxyuridylate (dUMP) and 5,10-CH2-THF as a cofactor; thus, its inhibition blocks DNA synthesis due to a lack of deoxythymidine 5’-triphosphate (dTTP), which slows tumor cell proliferation.

Folic acid and its derivatives are composed of a pteridine ring, p-aminobenzoic acid, and a glutamate residue [18]. Folic acid can enter healthy human cells through three different mechanisms: the reduced folate transporter (RFC), the proton-coupled folate transporter (PCFT), and specific folate receptors (FR) that have four isoforms (FRα, FRβ, FRγ, and FRδ) [19]. The folate receptors FRα, FRβ, and FRδ are found on the cell surface anchored by a glycosylphosphatidylinositol (GPI) while FRγ, which lacks a signal for GPI modification, is secreted to the extracellular region [20,21]. By coordinating folic acid or folate reduced to an RF, the complex (cell-surface receptor/ligand complex) is introduced into the cell by endocytosis [22].

Raltitrexed (RTX) can enter cells through two routes: the membrane transporter RFC (the main pathway for folate entry into cells) or by specifically binding to the α-folate receptor. The FRα receptor is a peripheral glycoprotein anchored to caveolins in the plasma membrane of cells. Once attached to the receptor, it will be introduced into the cell by receptor-mediated endocytosis. It is then encapsulated in an endosome that will fuse with a lysosome where the pH is roughly five. Finally, RTX is released into the cytosol where it polyglutamates and inhibits thymidylate synthase (TS).

The RFC carrier is the main route of entry of folates into cells. It is ubiquitously expressed in the body both in healthy and carcinogenic tissues [18]. However, the expression of FRα receptors in healthy tissues is limited to specific epithelial tissues (uterus, placenta, retina, kidneys, and choroid plexus); this receptor is overexpressed in most cancer cells especially in ovarian, cervical, pulmonary, renal, pancreatic, colorectal, cerebral, endometrial, chest, lung, and bladder cancers [23]. Therefore, FRα is a promising candidate for the diagnosis and treatment of a wide variety of cancer diseases.

Here, silver and gold nanoparticles with a size between 15 and 20 nm in diameter have been prepared and later functionalized with RTX. These nanoparticles can hopefully take advantage of one or both mechanisms of transport and targeting: passive and active targeting.

Passive targeting takes advantage of the fundamental differences between healthy and tumor tissues: The latter is characterized by a prolific leaky vasculature between the blood vessels surrounding the tumor [24] as a consequence of the angiogenesis, which is driven by the metabolic demands of cancer cells [25]. The presence of numerous large pores between 100 and 780 nm in diameter, (the pores in healthy tissue are less than 6 nm in diameter [26]), absence of effective lymphatic drainage, and an acidic microenvironment in the tumor cell environment due to its high glycolytic activity [27]. This is known as the enhanced permeability and retention (EPR) effect; it causes nanoparticles to accumulate, predominantly, in tumor cells.

Active targeting is based on the conjugation of the nanoparticle with a targeting motif that recognizes a component overexpressed by tumor cells thus allowing preferential accumulation of nanoparticles [24]. In our case, RTX is conjugated on the cell surface with folate receptors that are overexpressed on cancer cells by roughly 20-fold. While this is not the first time that the folic acid receptor has been used as a strategy to access cancer cells [28], we are not aware of any study in which precious metal nanoparticles were combined with RTX for possible cancer treatment with folate targeting.

## 2. Materials and Methods

### 2.1. Materials

All materials were of analytical-reagent grade and used without further purification. HAuCl_4_·3H_2_O, trisodium citrate, silver nitrate, NaBH_4_, *N*-(3-Dimethylaminopropyl)-*N*′-ethylcarbodiimide hydrochloride (EDC), dicyclohexylcarbodiimide (DCC), RTX were supplied by Sigma–Aldrich (Merck Life Science S.L.U., Madrid, Spain). Dimethyl sulfoxide (DMSO), ethanol, and methanol were from Scharlab (Scharlab, S. L., Barcelona, Spain). All solutions were prepared with deionized water using Milli-Q. Dialysis used SnakeSkin^®^ Dialysis Tubing—MWCO 3.500 g/mol (ThermoFisher, Scientific, Madrid, Spain).

### 2.2. Instrumentation

Infrared FTIR spectroscopy used a Bruker Tensor 27 instrument (Bruker Española, Madrid, Spain) in solid state. The ^1^H and ^13^C NMR spectra were recorded on a Bruker Advance Spectrometer (Bruker Española, Madrid, Spain) at 300 and 75 MHz at 23 °C. Ultraviolet-visible (UV-Vis) spectrum was obtained on a Shimadzu UV-2401PC Spectrometer (Shimadzu Europa GmbH, Duisburg, Germany). Dynamic light scattering (DLS) and zeta potential values were obtained on a Malvern ZetaSizer Nano ZS (Malvern Panalytical, Malvern, UK). Matrix-assisted laser desorption/ionization mass spectra (MALDI) was recorded with a Autoflex III MALDI TOF/TOF mass spectrometer provided with a Smartbeam Laser at 200 Hz (Bruker Española, Madrid, Spain). Functionalization of AgNP used a Biotage Initiator Classic microwave synthesizer (Biotage, NASDAQ, Stockholm, Sweden) at 400 W.

The size of gold and silver nanoparticles were determined by JEOL JEM 2010 transmission electron microscope (TEM) (JEOL Ltd., Tokyo, Japan) operated at 200 kV accelerating voltage. The sample for TEM measurements was prepared by dipping the TEM copper grid (400 mesh) in a dilute dispersion of nanoparticles in water.

### 2.3. Synthesis and Physicochemical Characterization of the Nanoparticles

**Scheme 1:**
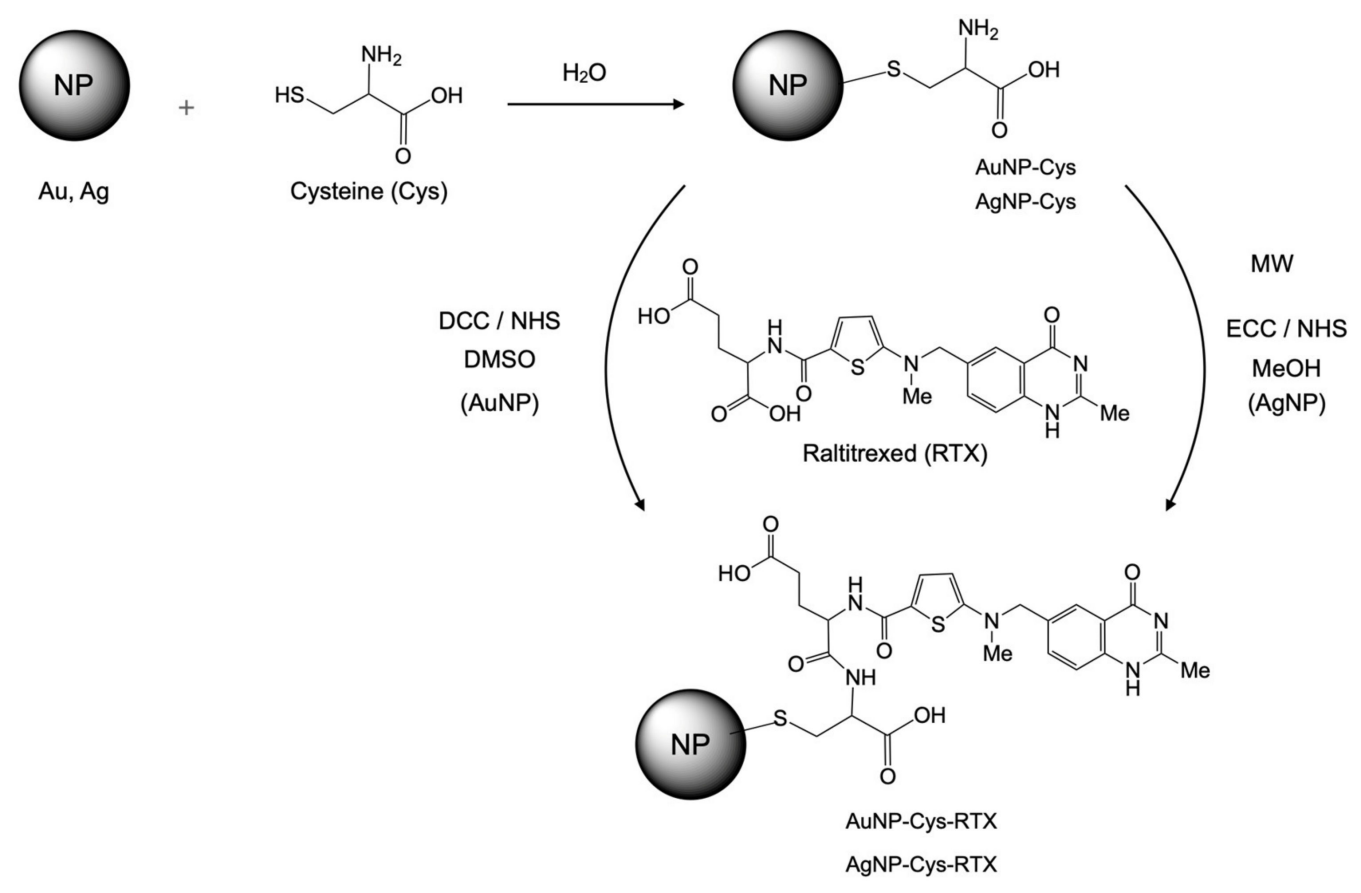
Synthesis of AuNP-Cys-RTX and AgNP-Cys-RTX.

The synthesis of AuNP-Cys-RTX and AgNP-Cys-RTX have been done according to the sequence presented in Scheme 1.

#### 2.3.1. Synthesis of Gold Nanoparticles AuNPs

AuNPs were synthesized through reduction of HAuCl_4_ by trisodium citrate [29]; 100 mL water was added into a flask and boiled. Next, 20 mg (5.08 × 10^−5^ mmol) HAuCl_4_ and 104.5 mg of trisodium citrate (3.6 × 10^−4^ mol; 1 mL 1%) were added rapidly into the boiling water successively. After boiling for 8 min with stirring, the color changed from colorless to red wine. The mixture was continuously stirred to room temperature and the volume was re-adjusted to 100 mL. The concentration was 58 μg/mL with a size of about 15 nm.

#### 2.3.2. Synthesis of AuNP-Cys

The previous solution of AuNPs was added rapidly to cysteine (6.16 mg, 5.08 × 10^−5^ mol in 1 mL of water) in four aliquots. The mixture was continuously stirred at room temperature for 24 h. The color changed from wine to dark grey-blue. The solution was then centrifuged and cleaned with water three times over 5 minutes. The product was then re-suspended in 10 mL in water, dialyzed for three days at room temperature and lyophilized for three days at −35 °C. The yield was 30%.

#### 2.3.3. Synthesis of AuNP-Cys-RTX

Here, 5 mg of RTX (0.01 mmol) was dissolved in 1 mL of DMSO. The solution was mixed with 2 mL of an aqueous solution of 15.5 mg of 1,3-dicyclohexylcarbodiimide (EDC) (0.075 mmol) and 1.7 mg of *N*-hydroxysuccinimide (NHS) (0.015 mmol). The AuNP-Cys (15.2 mg) were dissolved in 5 mL of H_2_O and were added into the solution above. The pH was adjusted to 8–9 with 1 M NaOH. The final suspension was stirred at 37 °C for 24 h. Finally, the nanoparticles were dialyzed for three days at room temperature and lyophilized for three days at −35 °C; 5.5 mg of the final product was obtained. Yield of AuNP functionalization: 64% The concentration of RTX covalently bound to the AuNP-Cys-RTX nanoparticles via amidation was estimated by elemental analysis (See Supplementary Materials).

#### 2.3.4. Synthesis of AgNP-Cys

AgNPs were synthesized through reduction of AgNO_3_ by sodium borohydride. To 29.5 mL of cysteine (12 mM) and 10 mL (1.0 mM) of AgNO_3_, we added a sodium borohydride solution dropwise (about 1 drop/second; 30 mL of 2 mM chilled in an ice bath). The mixture was continuously stirred on a magnetic stir plate. The color of the solution changed from colorless to dark brown. The mixture was continuously stirred two hours. The functionalized nanoparticles were precipitated by addition of ethanol. The solution was centrifuged and washed with ethanol three times (5 min at 8000 rpm). The product was then re-suspended in 10 mL in water dialyzed for three days at room temperature and lyophilized for three days at −35 °C.

#### 2.3.5. Synthesis of Silver Nanoparticles AgNP-Cys-RTX

The amidation reaction procedure was adapted from the literature [30] and used a microwave reactor. Here, 1.05 × 10^−5^ mmol of RTX (4.8 mg) were dissolved in 1 mL of distilled water; 13.3 mg of EDC and 2.18 mg of NHS were dissolved in 1 mL of distilled water. Both solutions were mixed, and the pH was adjusted to 8. Finally, 3.82 mg of AgNP-Cys were dispersed in MeOH (3 mL). This was added to the mixture, hermetically closed, and heated in the microwave reactor under magnetic stirring for 15 min at 140 °C and 4 bar. Finally, the nanoparticles were dialyzed for three days at room temperature and lyophilized for three days at −35 °C; 4.5 mg of the final product was obtained. The yield of AgNP functionalization was 89.4%. The concentration of RTX covalently bound to the AgNP-Cys-RTX nanoparticles via amidation was estimated by elemental analysis (See Supplementary Materials).

### 2.4. In Vitro Studies

#### 2.4.1. Cell Culture HCT116 Human Colorectal Carcinoma

The HCT116 human colorectal carcinoma cells were cultured in McCoy’s 5A medium (a medium rich in folic acid) with 10% FBS. To study cell viability, HCT116 cells were seeded in 96-well plates at a density of 5500 cells/well in 200 µL of McCoy 5A medium. After 24 h of incubation at 37 °C, the cells were treated for 24, 48, 72, and 96 h with free RTX (2000, 1000, and 250 nM); AuNP-Cys-RTX (quantity equivalent to 2000, 1000 and 250 nM RTX); or AuNP-Cys (amounts equivalent to AuNP-Cys-RTX). Due to low solubility, the samples were re-suspended in a H_2_O:DMSO (10:1) mixture, where the DMSO had a final concentration per well of 0.12% (*v/v*).

#### 2.4.2. Cell Culture A549 Human Lung Adenocarcinoma

Cells were obtained from the American Type Culture Collection (Manassas, VA). The cells were grown in RPMI 1640 medium supplemented with 2 mM L-glutamine (Sigma-Aldrich, Madrid, Spain), 10% (*v/v*) fetal bovine serum, 100 units per mL penicillin, and 100 μg mL^−1^ streptomycin. Tissue culture medium and supplements were purchased from LabClinics S.A. (Barcelona, Spain). When the cells reached 60–70% confluence, the vehicle, (RTX), AgNP-Cys, and AgNP-Cys-RTX were added to the medium for 24–96 h. Stock solutions of RTX were prepared at 1 mM in phosphate-buffered saline (PBS). Stock solutions of all nanoparticles were prepared at 1 mg Ag per mL in phosphate-buffered saline (PBS).

The A549 cells were plated in 96-well plates at a density of 4500 cells/well in 200 µL of growth medium without folic acid (Invitrogen, S.S., Barcelona, Spain) and grown for 24 h. To test the efficacy of compounds on cell growth, cells were exposed to RTX (25–1000 nM), AgNP-Cys, and AgNP-Cys-RTX (0.001–0.01 mg Ag per mL) for 24, 48, 72, or 96 h. The viability of the cells was measured after treatment.

#### 2.4.3. Cell Culture and Survival/Viability

The viability of cells was quantified by luminescence using the product CellTiter-Glo^®^ (Promega) based on the luciferase-catalyzed oxygenation reaction of luciferin in the presence of Mg^2+^, ATP, and O_2_. A higher cell viability led to higher ATP concentrations and thus more luminescence. Luminescence was quantified in a Synergy H1 luminometer BioTek^®^, (Agilent Technologies Spain, Las Rozas, Madrid).

The mean percentage of cell survival relative to that of vehicle-treated cells was calculated from data of three individual experiments performed in triplicate. The results are expressed as the mean ± S.E.M (Standard Error of the Mean). One-way analysis of variance (ANOVA) followed by a Newman–Keuls multiple comparison test was used for statistical evaluations. Differences were considered statistically significant at *p* < 0.05.

The amount of RTX in the AuNP-Cys-RTX and AgNP-Cys-RTX samples was estimated from the CHNS elemental microanalysis thereof (see Supplementary Materials). We calculated the percentage of RTX bound to nanoparticles. On the other hand, the amount of AuNP-Cys and AgNP-Cys was calculated from the weight of the AuNP-Cys-RTX and AgNP-Cys-RTX samples equivalent to 2000, 1000, and 250 nM of RTX.

## 3. Results

### 3.1. Characterization of Antifolate Hybrid Nanoparticles

#### 3.1.1. UV-Vis of AuNP-Cys-RTX

**Figure 1:**
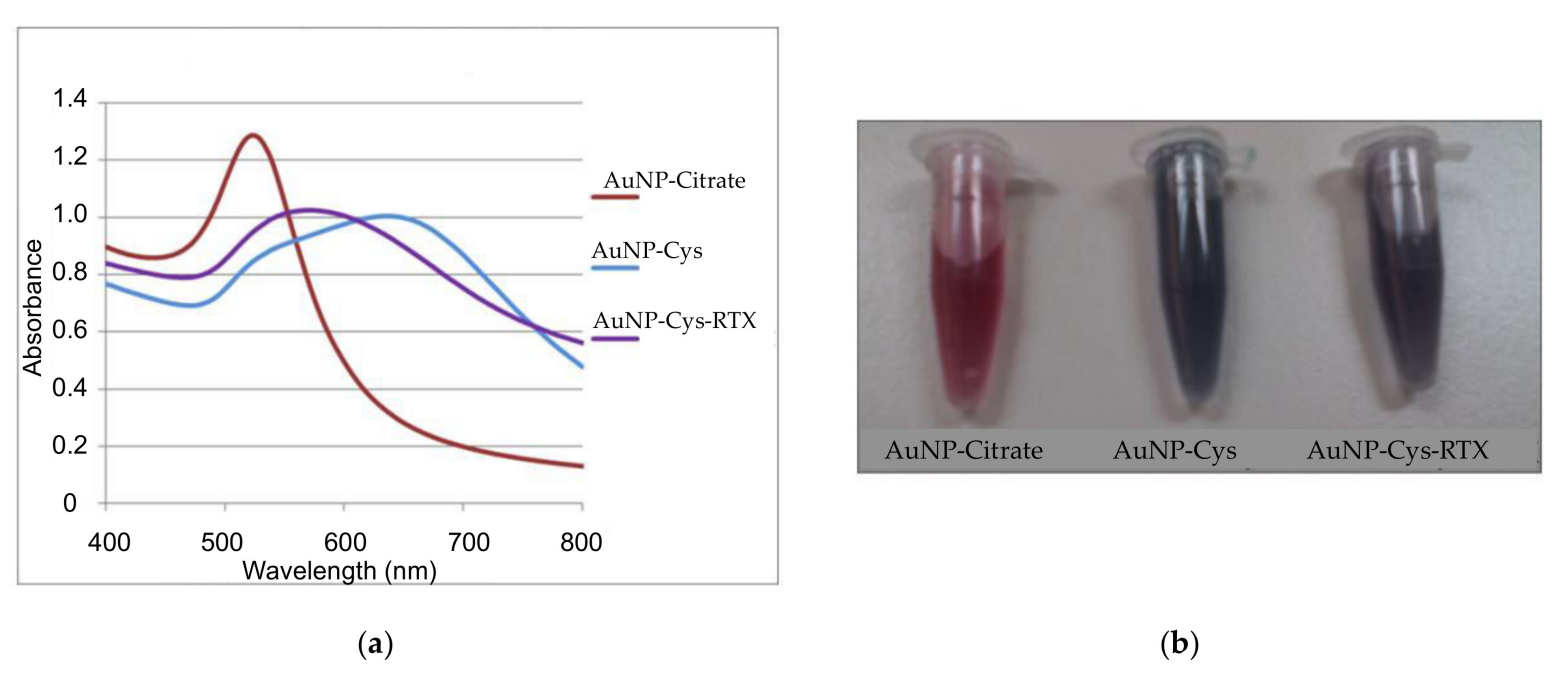
(**a**) UV–vis spectra of AuNP-citrate, AuNP-Cys, and AuNP-Cys-RTX. (**b**) shows the change in the color of AuNP-citrate after the addition of cysteine and Raltitrexed (RTX).

Figure 1 shows the different UV-vis spectra corresponding to the variation of the plasmonic band of the AuNPs corresponding to the three different stages leading to the preparation of the AuNP-Cys-RTX. After the preparation of AuNPs by reduction of HAuCl_4_ with sodium citrate, the plasmonic band (gold plasmon resonance) presents an absorbance maximum centered at 512 nm [31]. This band undergoes a broadening and bathochromic shift at 640 nm due to the exchange and functionalization of the AuNP surface with cysteine. This is due to the formation of new Au-S bonds between the Au of the nanoparticle and the thiol group of the cysteine. The AuNP-Cys formation creates a framework that will serve for the final amidation between a carboxylate group of the glutamine residue of RTX with the amino group of cysteine. This new functionalization causes a hypsochromic shift of the plasmon band at 520 nm. These three stages lead to different colors: AuNP-citrate red-wine (ruby-red); AuNP-Cys (blue), and AuNP-Cys-RTX (brown) due to electronic and colloidal changes in solution.

#### 3.1.2. FTIR of AuNP-Cys-RTX and AgNP-Cys-RTX

Figures S1–S3 show the FTIR of cysteine RTX, AuNP-Cys, AuNP-Cys-RTX, AgNP-Cys, and AgNP-Cys-RTX in a solid KBr matrix. The correct functionalization of the NPs is verified by observing how the thiol group of the cysteine located at 2550 cm^−1^ disappears when coordinated with the noble metal (Figures S1 and S2). This indicates that effective coordination occurred between cysteine and AuNP and AgNP. In parallel, the amidation reaction of AgNP-Cys and AuNP-Cys with RTX is verified in the FTIR by the disappearance of the C=O stretching band of AuNP-Cys (Figure S1) and AgNP-Cys (Figure S2) at approximately 1591 cm^−1^ as well as the appearance of the C=O stretching bands at 1625–1630 cm^−1^ of the amide (Figures S1 and S3) together with the amide bands at 1555-1560 cm^-1^. In addition, the area between 3500 and 3000 cm^−1^ is affected by the disappearance of the ammonium group of the cysteine (Figures S1 and S2) due to the presence of the new amide bond and the unaffected OH groups of the rest of RTX glutamate.

#### 3.1.3. Morphological Analysis of AuNP-Cys-RTX and AgNP-Cys-RTX

The shape and size distribution of the AuNP-Cys-RTX and AgNP-Cys-RTX nanoparticles (Figures S4 and S5) were analyzed by transmission electron microscopy (TEM). The AuNP-Cys-RTX have a gold core of 14.7 ± 0.4 nm. The AgNP-Cys-RTX have an average diameter of 20.4 ± 0.4 nm. In addition, these images show that the AuNP-Cys-RTX and the AgNP-Cys-RTX tend to reversibly aggregate when they are concentrated. The DLS studies indicated the presence of stable nanoparticles with a hydrodynamic diameter of 136 and 90 nm for AuNP-Cys-RTX and AgNP-Cys-RTX, respectively.

#### 3.1.4. Zeta Potential (ζ) of AuNP-Cys-RTX and AgNP-Cys-RTX

The deprotonation of carboxylic groups of both the cysteine and the carboxylate of the glutamate of the RTX caused the zeta potential to be −46.2 and −42.5 mV for AuNP-Cys-RTX and AgNP-Cys-RTX, respectively, at pH 7.4 and 30 °C. The results showed that these nanoparticles had an excellent dispersity (sample was 0.030 mg of metal (Au, Ag) per mL of each nanoparticle in a 0.01 M NaCl solution).

### 3.2. Cell Culture and Treatment for HCT 116 Cells with AuNP-Cys-RTX

**Figure 2:**
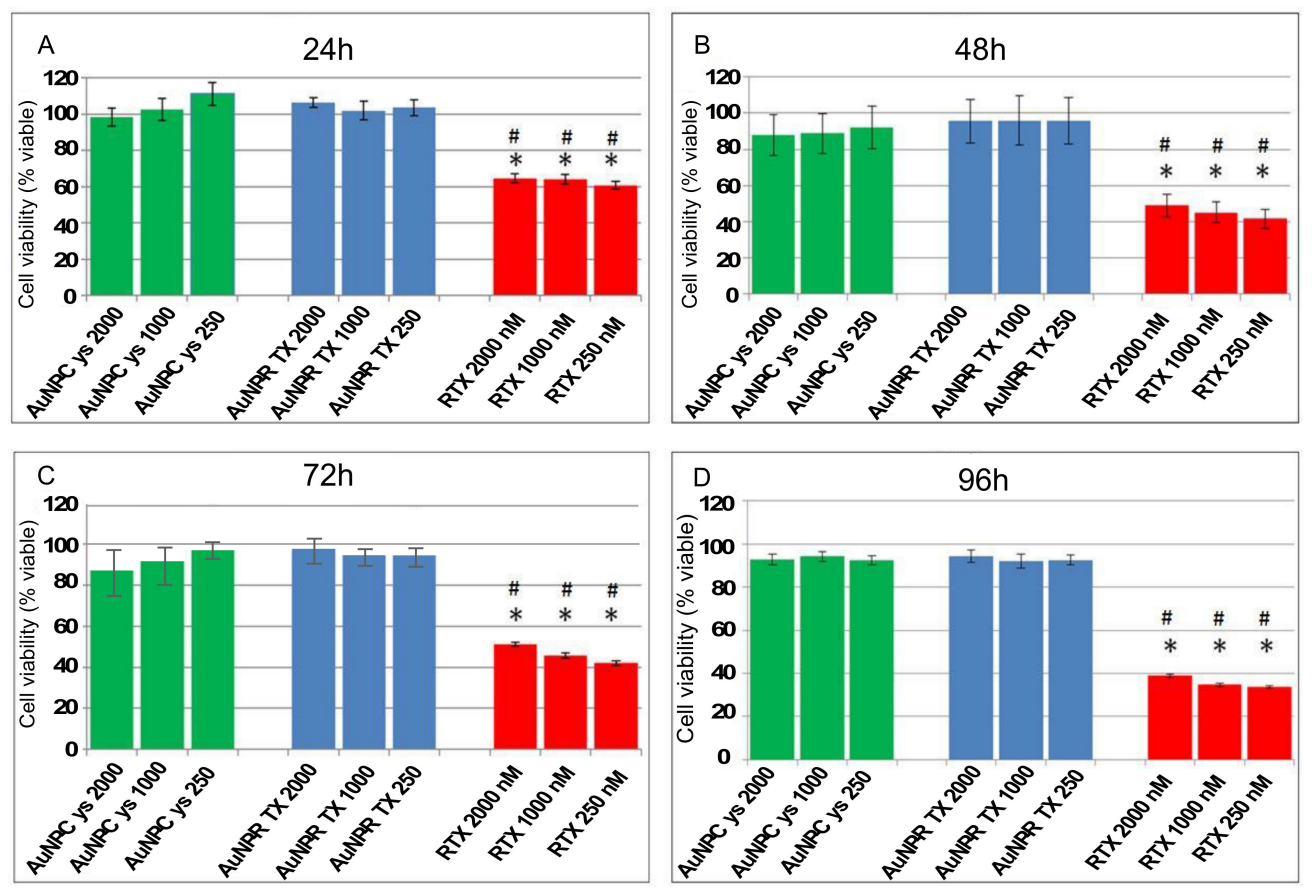
Behavior of the HCT116 cells after treatment with AuNP-Cys (green bar), AuNP-Cys-RTX (blue bar), and RTX (red bar) at different concentrations and time periods (**A**) 24 h, (**B**) 48 h, (**C**) 72 h, (**D**) 96 h. The nanoparticle (NP) concentrations are expressed as nM of RTX. Each bar represents the mean +/− SEM of three independent experiments performed in triplicate and normalized to non-treated cells (taken as 100%). The asterisk (*) and the hash mark (#) indicate significant differences between the viability resulting from treatment with “free” RTX and the equivalent concentrations of AuNP-Cys and AuNP-Cys-RTX, respectively (*p*-Value < 0.001).

Figure 2 shows that drug-bound nanoparticles (AuNP-Cys-RTX) do not seem to have the expected antineoplastic effect even after 96 h of treatment. On the other hand, cysteine nanoparticles (AuNP-Cys) do not present any cytotoxic effect. In this way, the antineoplastic action of RTX over HCT 116 cells is slow and reaches a maximum value after 96 h with 35% cell viability at 1000–250 nM (see Figure S6).

### 3.3. Cell Culture and Treatment for A549 Cells with AgNP-Cys-RTX

**Figure 3:**
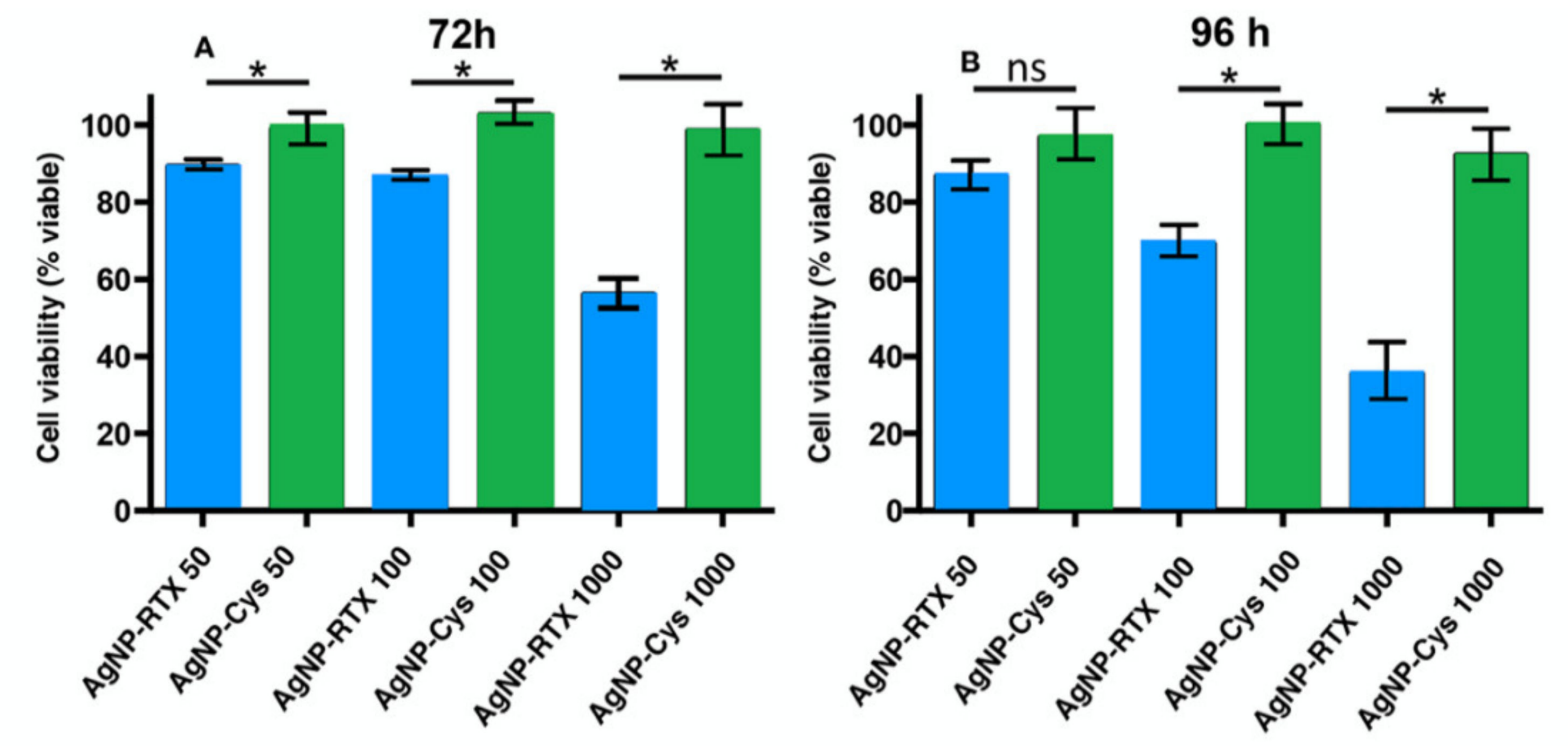
Behavior of the A549 cells after treatment with AgNP-Cys (green bar) and AgNP-Cys-RTX (blue bar) at different time periods (**A**) 72h (**B**) 96h and concentrations. The NP concentrations are expressed as nM of RTX. Each bar represents the mean +/− SEM of three independent experiments performed in triplicate normalized to non-treated cells (taken as 100%). Differences were analyzed preforming an ANOVA followed by a Newman–Keuls multiple comparison test. * *p*-value < 0.05; ns—no significant.

Figure 3 shows the variation in the cell viability of the AgNP-Cys and AgNP-Cys-RTX nanoparticles against the A549 tumor cells at different RTX concentrations (nM). The concentrations tested are between 50, 100, and 1000 and are monitored after 72 and 96 h. The results show a total compatibility of the A549 cells with the antifolate-functionalized nanoparticles; AgNP-Cys have no toxicity. The AgNP-Cys-RTX hybrid nanoparticles lead to a significant decrease in cell viability at 1000 nM for > 48 h. Thus, after 72 h, cell viability decreases by 52% and reaches a maximum decrease close to 38% after 96 h.

**Figure 4:**
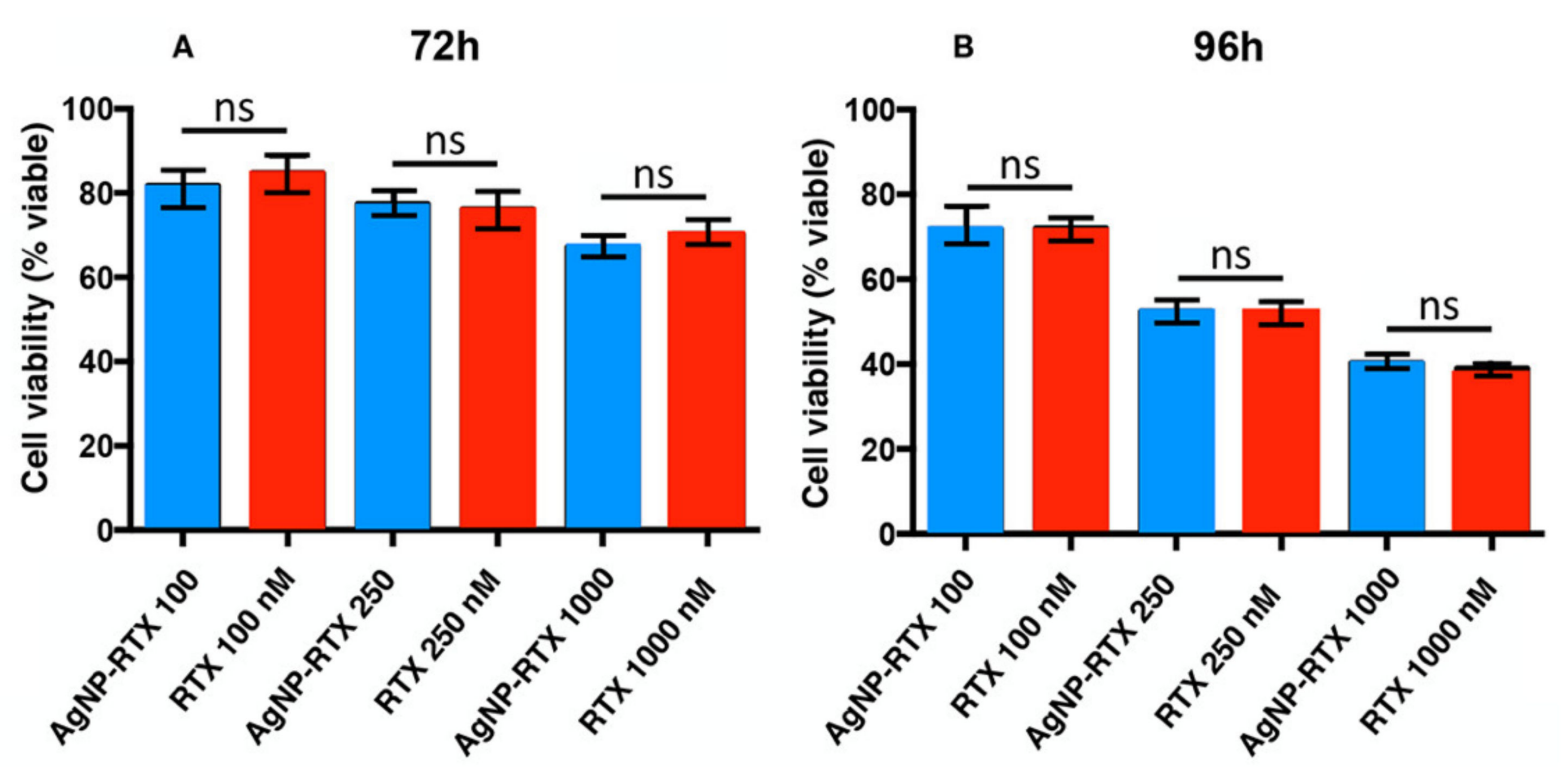
Behavior of the A549 cells after treatment with AgNP-Cys-RTX (blue bar) and RTX (red bar) at different time periods (**A**) 72 h (**B**) 96 h and concentrations. The NP concentrations are expressed as nM of RTX. Each bar represents the mean +/− SEM of three independent experiments performed in triplicate normalized to non-treated cells (taken as 100%). Differences were analyzed performing an ANOVA followed by a Newman–Keuls multiple comparison test. ns—no significant.

Figure 4 plots the viability data of cells treated with NP-RTX compared to the equivalent concentration of free RTX at 72 h and 96 h. The concentrations of AgNP-Cys-RTX shown here are calculated based on the equivalent concentration of free RTX.

## 4. Discussion

The anti-tumoral effect of the antifolate RTX transported into tumor cells through AuNP-Cys-RTX and AgNP-Cys-RTX nanoparticles was studied. The release mechanism of RTX inside the cell is due to the hydrolysis of the peptide bond via lysosomal proteases on the RTX conjugated in the nanoparticle; this process releases RTX in the cytoplasm.

Gold nanoparticles not functionalized with the drug (AuNP-Cys) have no cytotoxic effects. This agrees with the literature [32] confirming that non-functionalized gold nanoparticles are not toxic to cells [33].

However, the RTX-functionalized gold nanoparticles (AuNP-Cys-RTX) have no antineoplastic effects despite having introduced amounts equivalent to the three concentrations of free RTX studied. In fact, RTX bound to AuNP should be specifically recognized by folate receptors α (FRα) expressed on the plasma membrane of tumour cells allowing endocytosis of the nanoparticle and the subsequent release of the drug into the cell thanks to the breakdown of the amide bond between cysteine and RTX (breakdown mediated by acid pH and lysosome proteases).

To ensure that the loss of the RTX conjugation of the AuNPs had not occurred, the ^1^H-NMR spectrum of AuNP-Cys-RTX dispersed in DMSOd_6_:D_2_O was recorded after the dialysis purification process. The inset of Figure S7 shows the aromatic region of RTX in AuNP-Cys-RTX and the spectrum of free RTX dissolved in DMSO-d_6_.

After characterizing these nanomaterials, we also propose mechanisms impacting their action. For example, The HCT-116 cell line expresses a relatively low amount of folate α (FRα) receptors on its plasma membrane [34]. Several studies confirm that if this line is grown in a medium rich in folic acid (such as RPMI-1640 “standard” or McCoy 5A), then the expression of FRα can decrease up to 100% [35]. In our study, these cells were cultured in McCoy 5A medium, which explains the lack of antineoplastic potential evidenced in the viability test. The low amount of FRα and the high concentration of folic acid (which, in addition to inhibiting FRα expression, competes with the nanoparticle to bind to said receptor) are two important factors that can prevent internalization of AuNP-Cys-RTX in the cells.

This explains why free RTX has antineoplastic effects and not the nanoparticle. In addition to the FRα pathway, free drug can enter the cell via the RFC carrier, which is constitutively expressed on the HCT-116 cell membrane [36]. However, this carrier is too small to facilitate nanoparticle entry.

The data show that AuNP-Cys-RTX forms reversible aggregates (due to electrostatic forces) more than 100 nm in diameter. Although this effect would make it difficult for AuNP-Cys-RTX to enter cells, the literature recently showed how RTX forms irreversible aggregates with gold nanoparticles [37]. This could further worsen proper endocytosis and drug availability.

Quite the opposite occurs when RTX is used with silver nanoparticles for the treatment with cells that overexpress folate receptors (A549 human lung adenocarcinoma cells). 

Cell survival with both the AgNP-Cys-RTX hybrid material and the RTX drug is similar both at different concentrations and time periods. On one hand, this result is logical if one considers that the concentrations of nanoparticle and free RTX are identical. This result indicates that AgNP-Cys-RTX is at least as valid and effective as free RTX. It offers diffusion, vascular transfusion, and recognition by cellular FA receptors. The amide bond is broken inside the tumor cells to release conjugated drug. An in vivo treatment with these AgNP-Cys-RTX will likely produce fewer side effects because each AgNP-Cys-RTX transports more RTX (approximately 3000 per nanoparticle). Thus, a lower dose could achieve the same antitumor effectiveness [38].

## 5. Conclusions

This study evaluated two different functionalized nanoparticles: AuNP-Cys-RTX and AgNP-Cys-RTX. These were tested with A459 and HCT 116 cell lines in vitro with interesting conclusions. The first conclusion is that the RTX-functionalized AuNPs do not present sufficient dispersity to be used as a transport vehicle for RTX. This is because they aggregate due to the high affinity of RTX for AuNPs. However, cell lines, such as A549, that overexpress FA receptors can be used for active targeting. Future work will optimize the size and evaluate in vivo activity.

## Data Availability

Data sharing is not applicable to this article.

## Supplementary Materials

The following are available online at https://www.mdpi.com/1996-1944/14/3/534/s1, Figure S1: FTIR characterization of (1) AuNP-Cys, (2) AuNP-Cys-RTX, (3) Raltitrexed (RTX) and (4) Cysteine (Cys). Figure S2: FTIR characterization of (1) Cysteine (Cys) and (2) AgNP-Cys. Figure S3: FTIR characterization of (1) Raltitrexed (RTX) and (2) AgNP-Cys-RTX. Figure S4: TEM of AuNPs-Cys-RTX, and the statistical distribution of the size of the nanoparticles. Figure S5: TEM of AgNPs-Cys-RTX, and the statistical distribution of the size of the nanoparticles. Figure S6: Cell viability of HCT-116 after 72 and 96 hours of treatment with 2000, 1000 and 250 nM free Raltitrexed. Figure S7: ^1^H-RMN spectra of Raltitrexed in DMSOd_6_. Insert region shown the aromatic region of RTX supported on AuNP after lyophilization process.

## References

[1] Zhang L., Gu F.X., Langer R.S., Farokhzad O.C., Chan J.M., Wang A.Z. (2008). Nanoparticles in medicine: Therapeutic applications and developments. Clin. Pharmacol. Ther..

[2] Habibi N., Quevedo D.F., Gregory J.V., Lahann J. (2020). Emerging methods in therapeutics using multifunctional nanoparticles. WIREs Nanomed. Nanobiotechnol..

[3] Cheng K., Kang Q., Zhao X. (2020). Biogenic nanoparticles as immunomodulator for tumor treatment. WIREs Nanomed. Nanobiotechnol..

[4] Alavijeh A.A., Barati M., Barati M., Dehkordi H.A. (2019). The Potential of magnetic nanoparticles for diagnosis and treatment of cancer based on body magnetic field and organ-on-the-chip. Adv. Pharm. Bull..

[5] Kohler N., Sun C., Fichtenholtz A., Gunn J., Fang C., Zhang M. (2006). Methotrexate-immobilized poly(ethylene glycol) magnetic nanoparticles for MR imaging and drug delivery. Small.

[6] Bucharskaya A., Maslyakova G., Terentyuk G., Yakunin A., Avetisyan Y., Bibikova O., Tuchina E., Khlebtsov B., Khlebtsov N., Tuchin V. (2016). Towards effective photothermal/photodynamic treatment using plasmonic gold nanoparticles. Int. J. Mol. Sci..

[7] Wadajkar A.S., Dancy J.G., Carney C.P., Hampton B.S., Ames H.M., Winkles J.A., Woodworth G.F., Kim A.J. (2019). Leveraging Surface Plasmon Resonance to Dissect the Interfacial Properties of Nanoparticles: Implications for Tissue Binding and Tumor Penetration. Nanomedicine.

[8] Kang B., Mackey M.A., El-Sayed M.A. (2010). Nuclear targeting of gold nanoparticles in cancer cells induces DNA damage, causing cytokinesis arrest and apoptosis. J. Am. Chem. Soc..

[9] Vigderman L., Zubarev E.R. (2013). Therapeutic platforms based on gold nanoparticles and their covalent conjugates with drug molecules. Adv. Drug Deliv. Rev..

[10] Vivero L., Sendra J., Parkkola H., Querol J., Ramis M. (2011). Controlled-Release Nanoparticle System Comprising Hyaluronic Acid and Metal Nanoparticle Conjugate and Its Preparation. U.S. Patent.

[11] Mukhopadhyay D., Mukherjee P., Spaller M. (2010). Peptides Targeted GAIP-Interacting Protein GIPC and Nanoparticles for Therapeutic and Diagnostic Applications. U.S. Patent.

[12] Kim J.H., Choi Y.J. (2018). Anti-Cancer Adjuvant Composition Containing Silver Nanoparticles. U.S. Patent.

[13] López K.A., Piña M.N., Alemany R., Vögler O., Barceló F., Morey J. (2014). Antifolate modified iron oxide nanoparticles for targeted cancer therapy: Inclusion vs. covalent union. RSC Adv..

[14] Li Z., Mu Y., Peng C., Lavin M.F., Shao H., Du Z. (2020). Understanding the mechanisms of silica nanoparticles for nanomedicine. Wires Nanomed Nanobiotechol..

[15] Kholer N., Sun C., Wang J., Zhang M. (2005). Methotrexate-Modified Superparamagnetic nanoparticles and their intracellular uptake into human cancer cells. Langmuir.

[16] Cragg G.M., Grothaus P.G., Newman D.J. (2009). Impact of natural products on developing new anti-cancer agents. Chem. Rev..

[17] Jackman A.L., Taylor G.A., Gibson W., Kimbell R., Brown M., Calvert A.H., Judson I.R., Hughes L.R. (1991). ICI D1694, a quinazoline antifolate thymidylate synthase inhibitor that is a potent inhibitor of L1210 tumor cell growth in vitro and in vivo: A new agent for clinical study. Cancer Res..

[18] Gonen N., Assaraf Y.G. (2012). Antifolates in cancer therapy: Structure, activity and mechanisms of drug resistance. Drug Resist. Updates.

[19] Lederman J.A., Canevari S., Thigpen T. (2015). Targeting the folate receptor: Diagnostic and therapeutic approaches to personalize cancer treatments. Ann. Oncol..

[20] Shen F., Wu M., Ross J.F., Miller D., Ratnam M. (1995). Folate Receptor Type. Gamma. Is Primarily a Secretory Protein Due to Lack of an Efficient Signal for Glycosylphosphatidylinositol Modification: Protein Characterization and Cell Type Specificity. Biochemistry.

[21] UniProt. https://www.uniprot.org/uniprot/P41439.

[22] Zhao R., Diop-Bove N., Visentin M., Goldman I.D. (2011). Mechanisms of membrane transport of folates into cells and across epithelia. Annu. Rev. Nutr..

[23] Cho K., Wang X., Nie S., Chen Z.G., Shin D.M. (2008). Therapeutic nanoparticles for drug delivery in cancer. Clin. Cancer Res..

[24] Zwicke G.L., Mansoori G.A., Jeffery C.J. (2012). Utilizing the folate receptor for active targeting of cancer nanotherapeutics. Nano Rev..

[25] Wang J., Lu Z., Gao Y., Wientjes M.G., Au J.L.-S. (2011). Improving delivery and efficacy of nanomedicines in solid tumors: Role of tumor priming. Nanomedicine (London).

[26] Vander Heiden M.G., DeBerardinis R.J. (2017). Understanding the Intersections between Metabolism and Cancer Biology. Cell.

[27] Cerqueira B.B., Lasham A., Shelling A.N., Al-Kassas R. (2015). Nanoparticle therapeutics: Technologies and methods for overcoming cancer. Eur. J. Pharm. Biopharm..

[28] Marchetti C., Palaia I., Giorgini M., De Medici C., Iadarola R., Vertechy L., Domenici L., Di Donato V., Tomao F., Muzii L. (2014). Targeted drug delivery via folate receptors in recurrent ovarian cancer: A review. Onco Targets Ther..

[29] Herizchi R., Abbasi E., Milani M., Akbarzadeh A. (2014). Current methods for synthesis of gold nanoparticles. Artif. CellsNanomed. Biotechnol..

[30] Gutiérrez M.S., Piña M.N., Morey J. (2017). Fast microwave-assisted conjugation of magnetic nanoparticles with carboxylates of biological interest. RSC Adv..

[31] Patil V., Malvankar R.B., Sastry M. (1999). Role of particle size in individual and competitive diffusion of carboxylic acid derivatized colloid gold particles in thermally evaporated fatty amine films. Langmuir.

[32] Connor E.E., Mwamuka J., Gole A., Murphy C.J., Wyatt M.D. (2005). Gold nanoparticles are taken up by human cells but do not cause acute cytotoxicity. Small.

[33] Zeng S., Baillargeat D., Ho H.-P., Yong K.-T. (2014). Nanomaterials enhanced surface plasmon resonance for biological and chemical sensing applications. Chem. Soc. Rev..

[34] O’Shannessy D.J., Davis D.W., Anderes K., Somers E.B. (2016). Isolation of circulating tumor cells from multiple epithelial cancers with ApoStream(^®^) for detecting (or Monitoring) the expression of folate receptor alpha. Biomark. Insights.

[35] Hayashi I., Sohn K.-J., Stempak J.M., Croxford R., Kim Y.-I. (2007). Folate deficiency induces cell-specific changes in the steady-state transcript levels of genes involved in folate metabolism and 1-carbon transfer reactions in human colonic epithelial cells. J. Nutr..

[36] Morales C., Ribas M., Aiza G., Peinado M.A. (2005). Genetic determinants of methotrexate responsiveness and resistance in colon cancer cells. Oncogene.

[37] Lan W., Tan Q., Qiao J., Shen G., Qi L. (2019). D-Proline capped gold nanoclusters for turn-on detection of serum Raltitrexed. Chin. Chem. Lett..

[38] Liao J., Jia Y., Wu Y., Shi K., Yang D., Li P., Qian Z. (2020). Physical-, chemical-, and biological-responsive nanomedicine for cancer therapy. Wires Nanomed. Nanobiotechnol..

