# Supplementary material for: Raltitrexed-Modified Gold and Silver Nanoparticles for Targeted Cancer Therapy: Cytotoxicity Behavior In Vitro on A549 and HCT-116 Human Cancer Cells
Source: Materials (Basel). 2021 Jan 22;14(3):534. doi: 10.3390/ma14030534 (PMC7866044; doi:10.3390/ma14030534)
Supplement: Supplementary file 1 [file materials-14-00534-s001.pdf]

materials
MDPI

Supplementary Material

Raltitrexed-Modified Gold and Silver Nanoparticles for Targeted Cancer Therapy: Cytotoxicity Behavior in Vitro on A549 and HCT-116 Human Cancer Cells

Jeroni Morey[1], Pere Llinás[2], Alberto Bueno-Costa[2], Alberto J. León[1] and M. Nieves Piña.[1,*]

[1] Department of Chemistry, University of the Balearic Islands, Crta. de Valldemossa, Km. 7.5, 07122 Palma de Mallorca, Balearic Islands, Spain
[2] Department of Biochemistry, University of the Balearic Islands, Crta. de Valldemossa, Km. 7.5, 07122 Palma de Mallorca, Balearic Islands, Spain
* Correspondence: neus.pinya@uib.es; Tel.: +34-971-172847

1. Elemental Analysis

The CHNS Elemental Microanalyses were performed in the Research Assistance Center (Complutense University of Madrid, Spain), from 2.1 mg of AuNP-Cys-RTX and 2.3 mg lyophilized AgNP-Cys-RTX samples.

AuNP-Cys-RTX

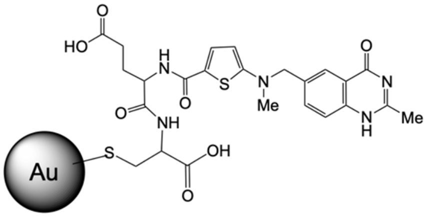

Element	% mass	% mol
Carbon	4.97	0.414
Hydrogen	0.96	0.952
Nitrogen	0.98	0.070
Sulfur	1.40	0.044

AgNP-Cys-RTX

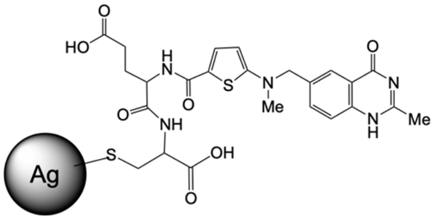

Element	% mass	% mol
Carbon	5.03	0.083
Hydrogen	1.49	1.478
Nitrogen	1.47	0.105
Sulfur	1.51	0.047

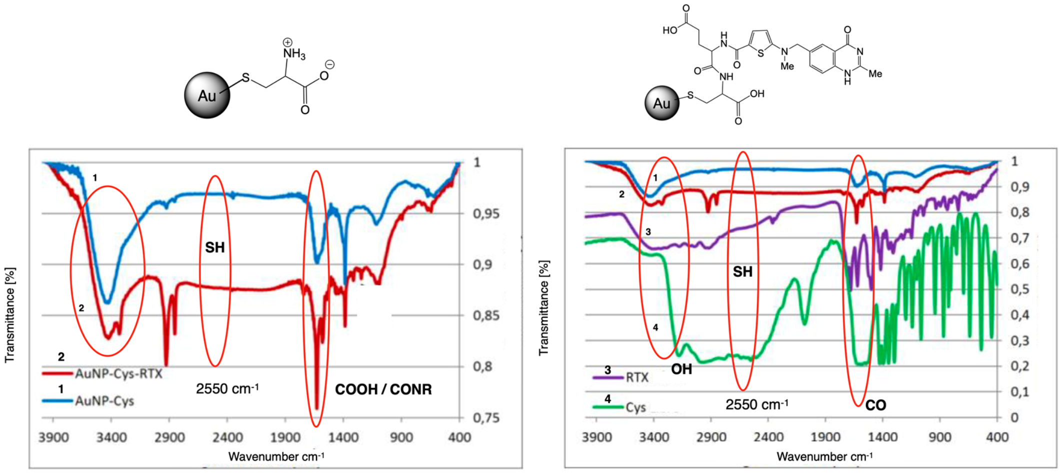

Figure S1. FTIR characterization of (1) AuNP-Cys, (2) AuNP-Cys-RTX, (3) Raltitrexed (RTX) and (4) Cysteine (Cys).

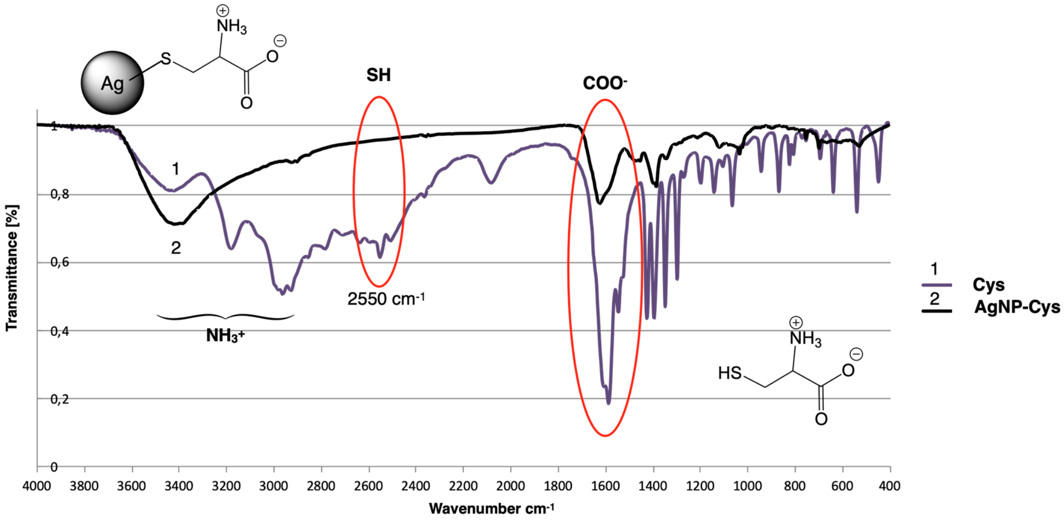

Figure S2. FTIR characterization of (1) Cysteine (Cys) and (2) AgNP-Cys.

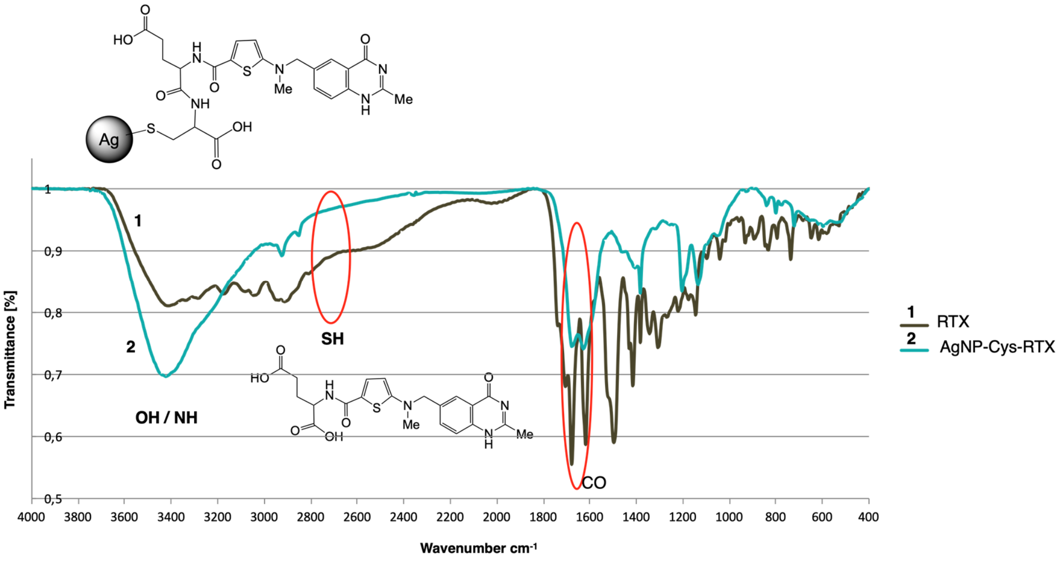

Figure S3. FTIR characterization of (1) Raltitrexed (RTX) and (2) AgNP-Cys-RTX.

2. Yield of AuNP functionalization:

To calculate the yield of the functionalization of the nanoparticle, we must take into account that AuNP can have cysteine remain molecules.

The cysteine molecules have 1 Sulfur and 1 Nitrogen atom (ratio N / S = 1).

The Cys-RTX molecules have 2 Sulfur and 5 Nitrogen atoms (ratio N / S = 2.5).

In this way, dividing (% Mol Element / Atomic Weight, see Elemental Analysis) of N (0.070) by that of S (0.044), we will obtain that the N / S ratio of AuNP-Cys-RTX sample is 1.59.

$$\% \text{ Functionalization Yield} = (1.59 / 2.5)\ 100\% = \mathbf{64\%} \quad (1)$$

This result indicates that for every 100 cysteine molecules bound to an AgNP, 64 of these molecules are bound, respectively, with a Raltitrexed molecule (Cyst-RTX).

To calculate the approximately theoretical number of Cys-RTX branches in AuNPs, the AuNP and gold atoms are assumed to be perfect spheres packed into the AuNP with a density of 64% (density resulting from the random close packing of perfect spheres of equal size).

The volume of the sphere is assumed to be: 4/3 (π)(r^3), and the radius of AuNP is (14.7 / 2) nm, and the radius of Au is 0.135 nm.

$$\textbf{Number Au Atom per AuNP} = (\% \text{ density Au Packet}) \times [(\text{Volume AuNPs})] / (\text{Volume Au})] = 0.64 \times [\ 4/3\ (\pi)\ (14.7/2)^3\ ] / [\ 4/3\ (\pi)\ (0.135)^3\ ] = \textbf{103286 atom Au/AuNP} \quad (2)$$

$$\textbf{Number S Atom per AuNP} = (\text{Number Au Atom per AuNP}) \times \text{Molar ratio of (S atom mol / Au atom mol)} = (\text{Number Au Atom per AuNP}) \times [\ (\%S / \text{atom weight S}) / (\%\ Au / \text{atom weigh Au})] = 103286 \times (0.044 / 0.435) = \textbf{10447 atom S / AuNP} \quad (3)$$

$$\textbf{\% Branches Cys-RTX =} \ \% \text{ Yield} \times \text{atom S} / (\text{AuNP} / 2) = 0.64 \times (10447 / 2) = \textbf{3343 branches per Cys-RTX / Au NP.} \quad (4)$$

3. Yield of AgNP functionalization:

In the same way as before:

Dividing (% Mol Element / Atomic Weight, see Elemental Analysis) of N (0.105) by that the S (0.047), we will obtain that the N / S ratio of AuNP-Cys-RTX sample is 2.234.

$$\% \text{ Functionalization Yield} = (2.234 / 2.5)\ 100\% = \mathbf{89.4\%} \quad (5)$$

This result indicates that for every 100 cysteine molecules bound to an AgNP, 89 of these molecules are bound, respectively, with a Raltitrexed molecule (Cyst-RTX).

$$\textbf{\% Branches Cys-RTX = 4100 branches Cys-RTX / AgNPs.} \quad (6)$$

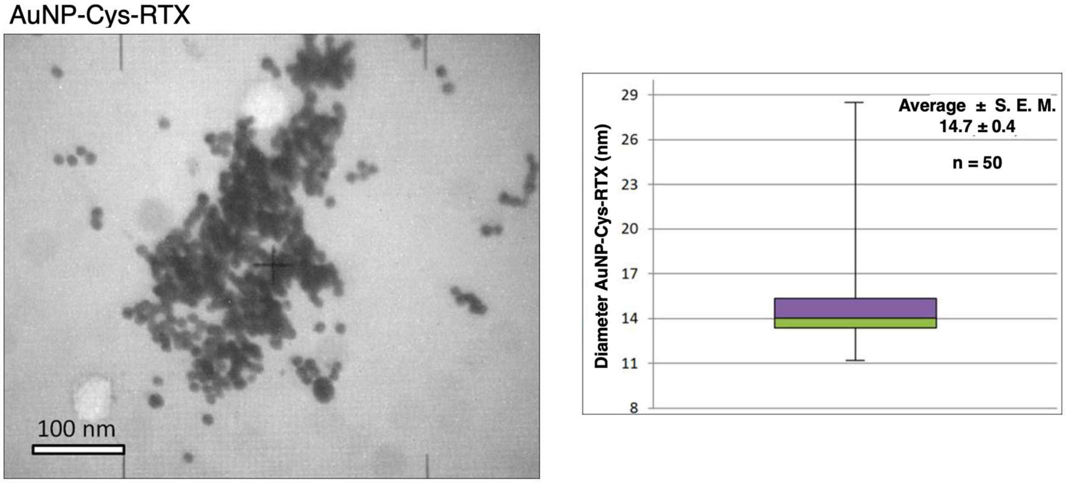

Figure S4. TEM of AuNPs-Cys-RTX, and the statistical distribution of the size of the nanoparticles. The box points out the nanoparticles distributed between the second and third quartile, while the bars indicate the values of the first and fourth quartiles.

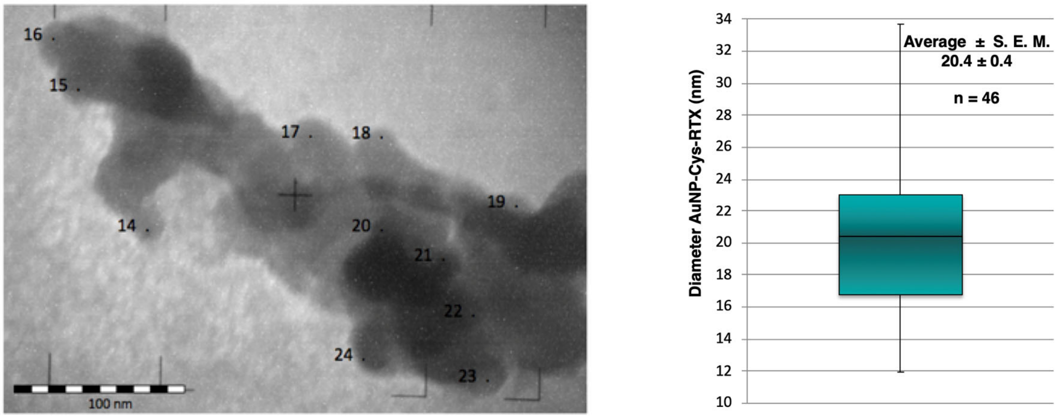

Figure S5. TEM of AgNPs-Cys-RTX, and the statistical distribution of the size of the nanoparticles. The box points out the nanoparticles distributed between the second and third quartile, while the bars indicate the values of the first and fourth quartiles.

AuNP-Cys-RTX

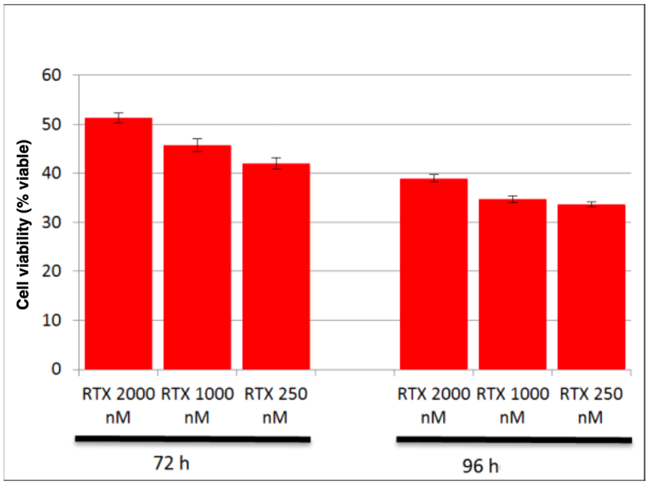

Figure S6. Cell viability of HCT-116 after 72 and 96 hours of treatment with 2000, 1000 and 250 nM free Raltitrexed. (*P*-Value < 0.0001).

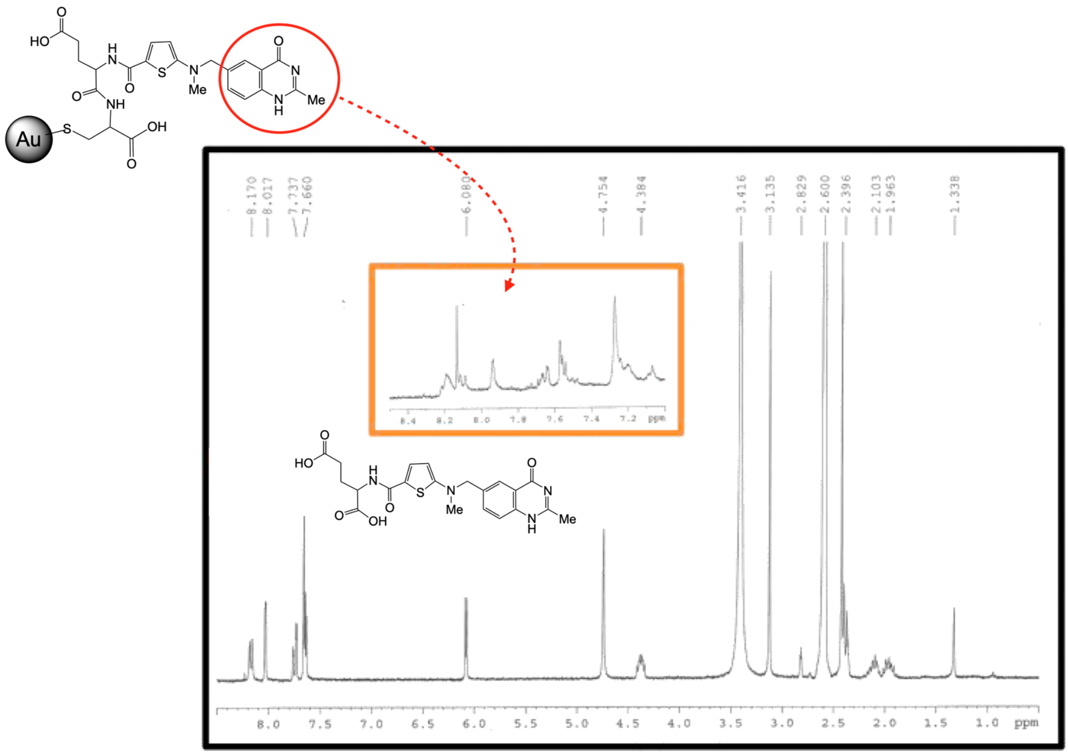

Figure S7. ^{1}H-NMR spectra of Raltitrexed in DMSO *d6*. The inset figure shows the RTX aromatic zone supported in AuNP after the lyophilization process.
